# Prenatal Lead Exposure and Weight of 0- to 5-Year-Old Children in Mexico City

**DOI:** 10.1289/ehp.1003184

**Published:** 2011-06-29

**Authors:** Myriam Afeiche, Karen E. Peterson, Brisa N. Sánchez, David Cantonwine, Héctor Lamadrid-Figueroa, Lourdes Schnaas, Adrienne S. Ettinger, Mauricio Hernández-Avila, Howard Hu, Martha M. Téllez-Rojo

**Affiliations:** 1 Department of Nutrition, Harvard School of Public Health, Boston, Massachusetts, USA; 2 Department of Environmental Health Sciences, and; 3 Department of Biostatistics, University of Michigan School of Public Health, Ann Arbor, Michigan, USA; 4 Division of Statistics, Center for Surveys and Evaluation Research, National Institute of Public Health, Cuernavaca, Morelos, México; 5 Division of Research on Public Health, National Institute of Perinatology, Mexico City, México; 6 Department of Environmental Health, Harvard School of Public Health, Boston, Massachusetts, USA; 7 Channing Laboratory, Department of Medicine, Brigham and Women’s Hospital, Harvard Medical School, Boston, Massachusetts, USA; 8 Department of Chronic Disease Epidemiology, Yale School of Public Health, New Haven, Connecticut, USA; 9 Ministry of Health, Distrito Federal, México

**Keywords:** bone lead, growth, weight

## Abstract

Background: Cumulative prenatal lead exposure, as measured by maternal bone lead burden, has been associated with smaller weight of offspring at birth and 1 month of age, but no study has examined whether this effect persists into early childhood.

Objective: We investigated the association of perinatal maternal bone lead, a biomarker of cumulative prenatal lead exposure, with children’s attained weight over time from birth to 5 years of age.

Methods: Children were weighed at birth and at several intervals up until 60 months. Maternal tibia and patella lead were measured at 1 month postpartum using *in vivo* K-shell X-ray fluorescence. We used varying coefficient models with random effects to assess the association of maternal bone lead with weight trajectories of 522 boys and 477 girls born between 1994 and 2005 in Mexico City.

Results: After controlling for breast-feeding duration, maternal anthropometry, and sociodemographic characteristics, a 1-SD increase in maternal patella lead (micrograms per gram) was associated with a 130.9-g decrease in weight [95% confidence interval (CI), –227.4 to –34.4 g] among females and a 13.0-g nonsignificant increase in weight among males (95% CI, –73.7 to 99.9 g) at 5 years of age. These associations were similar after controlling for concurrent blood lead levels between birth and 5 years.

Conclusions: Maternal bone lead was associated with lower weight over time among female but not male children up to 5 years of age. Given that the association was evident for patellar but not tibial lead levels, and was limited to females, results need to be confirmed in other studies.

The developmental origins hypothesis stipulates that constraints on fetal growth can have lasting impact on postnatal growth and related chronic diseases (Barker 2006; Eriksson et al. 1999). Although lead levels have decreased in the last few decades in the United States (Jones et al. 2009) and in other countries (Gulson et al. 2006; Hwang et al. 2004), environmental lead continues to be a public health problem that may restrict both fetal and child growth (Hernández-Avila et al. 2002). Maternal lead exposure during pregnancy is inversely related to infant size at birth (Gonzalez-Cossío et al. 1997; Jelliffe-Pawlowski et al. 2006). Prenatal lead exposure measured by maternal blood lead has been associated with decreases in children’s anthropometry at 6 and 15 months, respectively (Schell et al. 2009; Shukla et al. 1989, 1991).

The association of blood lead levels (pre- and postnatal) with child height has been more consistent than with weight (Andrews et al. 1994; Ong 2006; Peterson et al. 2004), although inferences from some studies have been limited by their cross-sectional design (Ballew et al. 1999; Frisancho and Ryan 1991; Kafourou et al. 1997; Schwartz et al. 1986). Less attention has been paid to prenatal lead exposure as measured by a cumulative marker of maternal lead burden and its relationship to child growth over time. Mobilization of lead in the bones is a source of endogenous exposure after external lead exposure has ceased (Gulson et al. 1997, 1999), especially during pregnancy and lactation, when the calcium needs of the developing fetus and infant are partially met by the mobilization of maternal calcium stores in the bone.

We have previously shown that maternal bone lead burden is associated with a decrease in birth weight (Gonzalez-Cossío et al. 1997) and lower weight gain from birth to 1 month of age and lower attained weight at 1 month (Sanín et al. 2001). However, no study has examined whether the effect of maternal bone lead concentration on child weight persists into early childhood. Numerous studies of determinants of child growth found sex-specific effects (Halileh et al. 2008; Hardy et al. 2004; Ogden et al. 2010), given different influences and patterns of growth and attained size at different ages in boys and girls. In addition, animal studies have demonstrated that hypothalamic–pituitary–gonadal axis regulation can be disrupted by lead (Dearth et al. 2002; Gorbel et al. 2002; Ronis et al. 1998).

In the present study we aimed to discern the longitudinal association of prenatal lead exposure, as measured by maternal bone lead burden, with child weight over time from birth to 5 years adjusted for postnatal lead exposure and stratified by sex. We hypothesized that prenatal lead exposure would lead to decreases in repeated measures of weight over time. In this study, we used maternal bone lead to assess prenatal lead exposure and concurrent child blood lead to assess postnatal lead exposure.

## Materials and Methods

*Study population.* The sample population consisted of three, sequentially enrolled longitudinal birth cohorts recruited by the same team of investigators between 1994 and 2005 at maternity hospitals serving low- to moderate-income populations in Mexico City. Although each cohort was studied for distinct specific aims, pooling was performed through the use of common methods for recruiting subjects and measuring lead exposure, covariates, and anthropometry, which were deliberately standardized.

Cohort 1 was a randomized control trial of the effect of calcium supplementation on blood lead levels during lactation (Gonzalez-Cossío et al. 1997; Hernández-Avila et al. 2003; Téllez-Rojo et al. 2002). Briefly, 617 mother–infant pairs were recruited between 1994 and 1995 and followed until 1997. The objective of cohort 2 was to understand the compartmentalization and mobilization of lead in plasma, whole blood, and bone during pregnancy. Cohort 2 comprised two groups, cohort 2A (*n* = 327) recruited either prepregnancy or at the first trimester of pregnancy and followed between 1997 and 2001, and cohort 2B (*n* = 462) recruited at delivery and followed between 1999 and 2003 (Hu et al. 2006; Téllez-Rojo et al. 2004). The 557 mothers in cohort 3 were enrolled in a randomized trial of calcium supplementation during pregnancy (Ettinger et al. 2009), and their children were followed between 2000 and 2007.

We applied similar exclusion criteria to the three cohorts, such as living outside of Mexico City or a physician’s diagnosis of current multiple pregnancies, gestational diabetes, or seizures (Ettinger et al. 2009; Gonzalez-Cossío et al. 1997; Téllez-Rojo et al. 2004). Of the initial 1,963 mothers originally recruited, 1,504 of their children were followed. For purposes of this study, we applied additional exclusion criteria to the 1,504 mother–infant pairs: missing information on child anthropometry at birth, date of birth, sex, or gestational age or maternal characteristics (*n* = 378 excluded). Also excluded were low-birth-weight (< 2,500 g, *n* = 77) and premature (< 37 weeks of gestation, *n* = 97) neonates, to minimize bias in the results due to postnatal catchup growth of these infants (Binkin et al. 1988). We excluded from further analysis subjects with extreme outliers (Rosner 1983) of child anthropometry (weight and height, *n* = 58), maternal anthropometry (calf circumference and height, *n* = 5), and lead measures (tibia and patella bone lead and log-transformed child blood lead, *n* = 34). Because participants could have been excluded for multiple reasons, the final analytic sample consisted of 1,000 mothers of 523 males and 477 females with complete information at baseline (cohort 1, 290; cohort 2A, 150; cohort 2B, 300; cohort 3, 260).

At time of recruitment, mothers were given detailed information about study procedures and provided information on ways to minimize lead exposure and signed a written letter of informed consent. The research protocol was approved by the ethics and research committees of the partnering institutions, including the National Institute of Public Health of Mexico, the Harvard School of Public Health, the Brigham and Women’s Hospital, the University of Michigan School of Public Health, and the participating hospitals.

*Study visits.* Participants were interviewed at delivery for all cohorts at the maternity hospitals and at the research clinic when the child was 1, 5, and 7 months of age (cohort 1) and at 3 and 6 months of age (cohorts 2A, 2B, and 3). Study visits were then scheduled at 12 months of age and every 6 months thereafter until 48 months of age for cohort 1 and 60 months of age for the other cohorts.

We estimated gestational age from the date of the last menstrual period recalled by the mother. At each study visit, we asked mothers whether they were breast-feeding. Because study visits were scheduled at different time points for each cohort, breast-feeding status was assessed at 7 months for cohort 1 and at 6 months for cohorts 2A, 2B, and 3. We refer to this variable as breast-feeding for 6 months.

*Anthropometry.* Weight and length of nude newborns were measured within 12 hr of delivery by experienced obstetric nurses using standardized procedures. At subsequent study visits, children’s weight and height were collected by trained staff using standard protocols (Habicht 1974; Lohman et al. 1988). Calibrated beam scales (model TD16; Oken, Naucalpan, México) were used and read to the nearest 10 g. Maternal height was obtained at 1-month postpartum using professional scales (model 420; BAME, Puebla, Mexico) read to the nearest millimeter. Maternal calf circumference was used as a proxy for maternal body size to control for maternal health and nutritional status because of incomplete information on the latter variable, consistent with previous research (Gonzalez-Cossío et al. 1997). Maternal calf circumference was measured at 1-month postpartum with plastic-covered fabric measuring tapes read to the nearest millimeter. Standardization exercises (Gonzalez-Cossío et al. 1997) were performed until the project staff reached imprecision errors (3 mm for calf circumferences and 2 mm for height) equal to or below those reported by Lohman et al. (1988).

*Exposure assessment.* Bone lead measurements. *In vivo* maternal bone lead measurements (micrograms of lead per gram of bone mineral) were taken at 1 month postpartum (± 5 days) at two bone sites, the midtibial shaft (cortical bone) and the patella (trabecular bone), to represent cumulative lead exposure. Mexican law forbids nonemergency radiologic procedures in women during pregnancy. Bone lead was measured noninvasively using a spot-source ^109^Cd K-shell X-ray fluorescence (K-XRF) instrument constructed at Harvard University and installed in a research facility in the American British Cowdray Medical Center in Mexico City. The physical principles, technical specifications, and validation of this and other similar K-XRF instruments have been described previously (Aro et al. 1994; Burger et al. 1990; Hu et al. 1989, 1991, 1995, 1998).

*Blood lead measurements.* Whole blood was collected from children in trace-metal–free tubes (BD Vacutainer® no. 367734; Becton-Dickinson, Franklin Lakes, NJ, USA), and sampling was conducted at each interview by trained staff using standard protocols. Blood lead samples were analyzed at the metals laboratory of the American British Cowdray Hospital in Mexico City using graphite-furnace atomic-absorption spectroscopy (model 3000; Perkin-Elmer, Chelmsford, MA, USA). The units of measurement are in micrograms per deciliter. The instrument precision is within 1 μg/dL, and the limit of detection is < 1 μg/dL.

External blinded quality-control samples were analyzed by the Maternal and Child Health Bureau (MCHB) and the Wisconsin State Laboratory of Hygiene (WSLH) Cooperative Blood Lead Proficiency Testing Program (PBPTP) and demonstrated adequate precision and accuracy determinations at the American British Cowdray Hospital (*r* = 0.99; mean difference of 0.17 mg/dL compared with MCHB and WSLH PBPTP blanks and spikes) (Gonzalez-Cossío et al. 1997).

Blood lead measures were not collected at every visit because of child or maternal refusal or the child’s inability to give blood, because the child was sick, or because a blood lead measure was not scheduled at that visit.

*Statistical analyses.* Univariate and bivariate analyses. Descriptive statistics and distributions were examined. We identified extreme outliers of maternal and child anthropometry and lead measures using the generalized extreme studentized deviation method (Rosner 1983). We carried out *t*-tests for continuous variables and chi-square test for categorical variables to compare differences between participants with complete information and participants excluded because of the additional study eligibility criteria. Age-specific weight- and length/height-for-age *z*-scores were calculated using the SAS macro based on the 2006 World Health Organization (WHO) growth reference (WHO 2006).

We chose covariates *a priori* based on biological relevance as known predictors of child’s weight or potential confounders of the association between prenatal lead exposure and child weight over time: aternal anthropometry (height and calf circumference), sociodemographic characteristics (education, marital status, parity, age at delivery), lifestyle (breast-feeding), calcium treatment group assignment during the randomized controlled trials, and child characteristics (e.g., gestational age, cohort, and repeated measures of child height) (Gonzalez-Cossío et al. 1998). We examined the correlations among pairs of predictors to assess potential multicollinearity. We performed simple linear regression models to examine the bivariate relationship between lead biomarkers. We defined statistical significance as *p* < 0.05.

Outcome models. Given the repeated measures of child weight over time, we used varying coefficient models with random effects to quantify the multivariable association of child, maternal, and lead characteristics with weight over time:

*y_i_*_(_*_t_*_)_ = β_0_(*t*) + β_1_(*t*)*X_i_* + *b_i_*(*t*) + ε*_it_*, [1]

where the attained weight by child *i* at age *t* is denoted by *y_i_*(*t*), β_0_(*t*) is the average weight for the sample population at time *t*, β_1_(*t*) represents changes to the average weight trajectory associated with covariate *X*, *b_i_*(*t*) is a subject-specific random effect, and ε*_it_* is the random error. The terms β_0_(*t*) and β_1_(*t*) are time-varying coefficients and are smooth functions of child’s age in months. For example, β_0_(*t*) evaluated at *t* = 0, β_0_(0), would be the weight at birth for the average individual; similarly, β_1_(0) would be the average difference in weight at birth associated with a one-unit increase in *X*. Hence, the model allows the association between weight and the covariate *X* to change with child’s age (i.e., a time-varying coefficient) and be evaluated at particular times during child’s growth (e.g., at 1 month after birth).

We define “weight trajectories” as the repeated measures of child’s attained weight at each age (in months) between birth and 5 years of age. The intercept and child’s age were treated as random effects. All other variables were fixed effects and were centered at their mean. We examined models separately for tibia and patella because of collinearity between the two bone lead measures; bone lead measures were standardized.

We analyzed data using SAS (version 9.2; SAS Institute Inc., Cary, NC, USA) and R (version 2.11.1; R Foundation for Statistical Computing, Vienna, Austria). We constructed the predicted weight trajectories for children 2 SDs above and below the sample mean of bone lead by estimating β^ˆ^_0_(*t*) ± 2β^ˆ^_lead_(*t*), which can be obtained from model output using the “predict” function in R.

## Results

At baseline, we found statistically significant differences in several characteristics between those participants included in the analyses (Table 1) and those excluded (based on observations with nonmissing data): Excluded children had a lower mean gestational age (38.0 vs. 39.2 weeks) and birth weight (3.0 vs. 3.2 kg), were more likely to be born to single mothers (12% vs. 7.5%), and were less likely to be breast-fed for 6 months (61.3% vs. 68.1%). Excluded children also had higher corresponding bone lead for maternal patella (12.9 vs. 10.4 μg/g) and tibia (10.2 vs. 8.7 μg/g), consistent with the exclusion of outlier observations from the study sample. Participating mothers had a mean age at delivery of 26 years. Most mothers were married (71%) and had on average ≥ 10 years of education. The correlation between the patella and tibia lead measures was 0.34 (*p* < 0.001) (Table 1). Bone lead levels differed by cohort (mean ± SD: cohort 1, 13.6 ± 14.5 μg/g; cohort 2A, 12.9 ± 11.1 μg/g; cohort 2B, 8.6 ± 9.9 μg/g; cohort 3, 7.2 ± 9.6 μg/g; but did not differ appreciably by offspring sex (males, 10.2 ± 11.9 μg/g; females, 10.7 ± 11.7 μg/g). Children’s mean blood lead level across all ages was 3.8 ± 2.9 μg/dL. Blood lead levels for 31% of children exceeded 10 μg/dL at some point in the study, but this accounted for only 8% of all blood lead measures. The males’ mean ± SD weight-for-age and length-for-age *z*-scores at 12 months were lower than the WHO reference (–0.21 ± 0.92 and –0.52 ± 0.97, respectively). Females were close to the mean for weight-for-age but shorter than the reference population (–0.00 ± 0.84 and –0.22 ± 0.96, respectively). At 48 months, both males and females weighed less and were shorter than the reference population.

**Table 1:** Characteristics of participants in combined cohort analysis and excluded participants.

Included*a* [*n* (%) or mean ± SD]	Excluded		
	Characteristic	*nb*	*n* (%) or mean ± SD
Child characteristics						
Male sex		523 (52.3)		502		243 (48.4)
Gestational age (weeks)		39.2 ± 1.1		429		38.0 ± 2.1*
Birth weight (kg)		3.2 ± 0.4		432		3.0 ± 0.6*
Cohort*c*				504*		
1		290 (29.0)				135 (26.8)
2A		150 (15.0)				103 (20.4)
2B		300 (30.0)				133 (26.4)
3		260 (26.0)				133 (26.4)
Maternal characteristics						
Age at delivery (years)		25.7 ± 5.3		436		25.6 ± 5.4
Calf circumference (cm)		34.1 ± 3.0		361		34.4 ± 3.8
Height (cm)		154.6 ± 5.7		421		154.0 ± 6.1
Marital status				433*		
Married		707 (70.7)				296 (68.4)
With partner		218 (21.8)				85 (19.5)
Single, separated, or divorced		75 (7.5)				52 (12.0)
Education (years)		10.5 ± 3.2		437		10.2 ± 3.1
Parity				438		
Primiparous		387 (38.7)				175 (40.0)
1 previous child		344 (34.4)				149 (34.0)
≥ 2 previous children		269 (26.9)				114 (26.0)
Smoked during pregnancy (%)		45 (4.5)		434		16 (3.7)
Breast-fed for 6 months (%)		681 (68.1)		501		307 (61.3)*
Lead biomarkers						
Child blood lead (μg/dL)		3.8 ± 2.9		347		3.8 ± 3.0
Patella (μg/g)		10.4 ± 11.8		258		12.9 ± 15.9*
Tibia (μg/g)		8.7 ± 9.7		254		10.2 ± 11.8*
**a**Sample size = 1,000 except for tibia (*n* = 846), and children’s mean blood lead level across all ages (*n* = 673). **b**Numbers of excluded observations with information available on each characteristic. A total of 504 observations were excluded. **c**The sample population consists of the combination of three sequentially enrolled longitudinal pooled birth cohorts. *p < 0.05.						

Table 2 shows the associations between maternal patella lead and other lead biomarkers. Patella bone lead was significantly positively associated with repeated measures of child blood lead through 48 months.

**Table 2:** Associations between maternal patella bone lead and other lead biomarkers.

Measure	Effect estimate	SE	*p*-Value
Repeated measures of child blood lead (µg/dL)*a*						
12 months		0.50		0.12		< 0.0001
24 months		0.39		0.10		0.0002
36 months		0.39		0.12		0.0011
48 months		0.54		0.14		0.0002
60 months		0.29		0.22		0.2014
Maternal tibia lead (µg/g)		0.42		0.03		< 0.0001
**a**Yearly blood lead measures at visits common to all three cohorts (except cohort 1, whose last study visit was at 48 months).						

**Figure 1:**
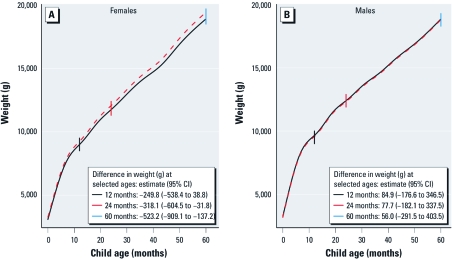
Adjusted predicted weight trajectories for females (*A*) and males (*B*) whose mothers had 2 SDs of patella lead above (solid black curves) and below the mean (dashed red curves), obtained using varying coefficient models with random effects to quantify the multivariable association of lead with weight over time. Smooth functions of child’s age in months were used. The weight trajectories are adjusted for cohort; maternal age at delivery, calf circumference, height, education, number of pregnancies, breast-feeding for 6 months, and calcium treatment group assignment; and child’s gestational age at birth and height. *p*-Value for testing the difference of effect across age among females = 0.08.

Figure 1 shows the adjusted weight trajectories of children whose maternal patella lead levels were 2 SD above (high exposed) and 2 SD below (low exposed) the sample mean, stratified by sex. Females with high lead levels had a reduced weight trajectory compared with females with low lead levels (Figure 1A). The association between patella lead and weight was not statistically significant until 19 months of age, when the mean weight difference between those exposed to high lead compared with those exposed to low lead was –280 g [95% confidence interval (CI), –570 to –3 g]. Between 19 months and 5 years of age, the association of high versus low maternal patella lead with weight became significantly larger for females (*p*-value for trend = 0.08; Figure 1A). Among males, the difference in estimated weight trajectories was not noticeably different between those exposed to low versus high maternal patella lead levels (Figure 1B).

Table 3 gives the associations between a 1-SD increase in maternal patella lead and child weight at various ages. After controlling for children’s concurrent blood lead levels, the associations described above were similar to the model without child’s blood lead for both sexes in that maternal patella lead was associated with a decrease in females’ but not males’ weight. In adjusted models, the association between maternal patella lead with weight of females was negative, increased monotonically with age, and became statistically significant at 15 months of age, when a 1-SD increase in maternal patella lead was associated with a –77.2-g decrease in weight (95% CI, –152.8 to –1.5 g). Among males, a 1-SD increase in maternal patella lead was associated with a slight decrease of 13.6 g by 48 months of age (95% CI, –97.9 to 70.8 g).

**Table 3:** Associations between a 1-SD increase in maternal patella lead (µg/g) and weight of females and males (g) at different ages [β (95% CI)].

Child age (months)	Unadjusted	Adjusted*a*	Fully adjusted*b*
Females						
Birth		–45.2 (–145.6 to 55.2)		–45.2 (–122.8 to 32.3)		–45.7 (–131.7 to 40.2)
12		–57.5 (–152.8 to 37.7)		–62.4 (–134.5 to 9.7)		–70.9 (–147.9 to 6.0)
24		–69.9 (–164.3 to 24.6)		–79.5 (–151.1 to –8.0)		–96.1 (–170.4 to –21.8)
36		–82.2 (–180.4 to 16.0)		–96.6 (–172.6 to –20.6)		–121.3 (–200.0 to –42.6)
48		–94.5 (–200.4 to 11.4)		–113.8 (–198.5 to –29.1)		–146.4 (–235.5 to –57.4)
60		–106.8 (–223.6 to 10.0)		–130.9 (–227.4 to –34.4)		–171.6 (–275.2 to –68.0)
Males						
Birth		27.2 (–60.1 to 114.5)		22.5 (–47.5 to 92.6)		72.3 (–9.8 to 154.4)
12		16.2 (–66.4 to 98.8)		20.6 (–44.7 to 86.1)		29.4 (–42.1 to 100.8)
24		5.2 (–76.9 to 87.2)		18.7 (–46.2 to 83.7)		27.8 (–43.5 to 99.1)
36		–5.8 (–91.6 to 80.0)		16.9 (–52.0 to 85.8)		7.9 (–67.3 to 83.1)
48		–16.8 (–110.1 to 76.5)		14.9 (–61.5 to 91.5)		–13.6 (–97.9 to 70.8)
60		–27.8 (–131.6 to 76.0)		13.0 (–73.7 to 99.9)		–35.0 (–132.4 to 62.3)
**a**Adjusted for cohort; maternal age at delivery, calf circumference, height, education, number of pregnancies, breast-feeding for 6 months, and calcium treatment group assignment; and child’s gestational age at birth and height. **b**Additionally adjusted for repeated measures of concurrent child blood lead.						

We found no evidence of an association between maternal tibia lead and weight in females at any time, although the direction of the results was negative (data not shown). Among males and in adjusted models, however, the direction of the association was positive at all ages and was significant from 36 to 60 months. At 36 months, a 1-SD increase in tibia lead was related to an 82.2-g increase in weight (95% CI, 8.2–156.1 g). After adding concurrent blood lead levels in the model, maternal tibia lead was no longer associated with weight of males.

We observed no change in the association between maternal patella lead and child weight over time among both girls and boys when we included the outliers of maternal patella lead (~ 4.5 SD above the mean) in the models (data not shown).

## Discussion

This is the first longitudinal study to investigate the association of cumulative prenatal lead exposure, as measured by maternal bone lead, with child’s weight between birth and 5 years of age. These results extend our previous findings (Gonzalez-Cossío et al. 1997; Sanín et al. 2001) relating prenatal lead exposure to weight decrements at birth and 1 month of age, by examining associations with weight up to 5 years. We found evidence of a sustained decrease in child weight over time correlated with lead concentration in one bone compartment (patella) and confined to one sex (females).

Cross-sectional studies have investigated the association of child blood lead levels with weight (Ignasiak et al. 2006; Little et al. 2009). Little et al. (2009) found that a 10-μg/dL increase in concurrent child blood lead was associated with a 1,900-g (95% CI, 1,700–2,100 g) decrease in weight among 360 children 2–12 years of age. In another longitudinal study, Schell et al. (2009) observed a decrease in weight-for-age *z*-score at 6 months (mean ± SE, –0.77 ± 0.34) but not 12 months of age associated with maternal second-trimester blood lead levels higher than the median (≥ 3 μg/dL).

Fetal lead exposure may exert its effect on growth through disruption of thyroid signaling. Specifically, a lead-induced deficiency in maternal thyroid hormone might limit the amount available to the fetus (Zoeller et al. 2002), thus decreasing soft tissue and organ growth (Glinoer 1997; Hernández-Avila et al. 2002). Maternal blood lead has been negatively associated with maternal free thyroxine among mothers with higher blood lead levels (mean ± SD, 20.5 ± 7.3 μg/dL) but not among those with lower blood lead levels (5.6 ± 1.9 μg/dL) (Lamb et al. 2008).

We did not find an association between maternal tibia lead with weight, perhaps because lead accumulates more slowly in cortical bone (tibia) than in trabecular bone (patella). Trabecular bone is more vascularized and reflects more recent lead exposure (Hu et al. 1989, 1998). However, we are not able to explain why maternal tibia lead was associated with increased weight among males in adjusted models.

We found differences by sex. Cross-sectional lead levels have been associated with decreases in weight and body mass index among females but not males between 7 and 14 years of age (Ignasiak et al. 2006). Lead might disrupt the endocrine system’s functions differently among males and females. The effect of lead on growth might be mediated by its impact on estrogen metabolism. Estrogen stimulates growth hormone secretion, which in turn leads to increased levels of insulin-like growth factor-1 (IGF-1) and body growth (Juul 2001; Leung et al. 2004). Prenatal lead exposure has been demonstrated to decrease levels of estradiol (Dearth et al. 2002; Iavicoli et al. 2004) and IGF-1 (Ronis et al. 1998) in animal studies. Because estradiol is found in larger concentrations in females, it is biologically plausible that growth suppression would have been more prominent in females through the role of estrogen on growth.

The decrease in maternal bone lead levels in our later cohorts reveals the reduction in environmental lead exposure, particularly due to phasing out of leaded gasoline in Mexico in the mid-1990s (Romieu et al. 1994). Bone lead levels in this study were comparable to those of a cohort of 700 women in Los Angeles in the middle to late 1990s where mean calcaneus lead (a measure of trabecular bone lead) was 10.7 ± 11.9 μg/g and tibia bone lead was 8.0 ± 11.4 μg/g (Rothenberg et al. 2002). However, mean bone lead levels in our study were higher than than those of a cohort of residents in southern Ontario, in which tibia lead levels among women 15–40 years of age were around 5 μg/g (average bone lead estimated from Gamblin et al. 1994, their Figure 2), or those of women who had recently delivered in Boston in the late 1980s (mean patella lead, 5 μg/g) (Hu et al. 1996). Children’s blood lead levels in our pooled analysis were higher than mean levels among 1- to 5-year-olds in the United States between 1999 and 2008 [Centers for Disease Control and Prevention (CDC) 2009]. Currently, no health policies have set a level of concern for maternal bone lead concentrations. This research adds to the epidemiologic evidence that maternal bone lead burden is associated with detrimental effects on child growth and development (CDC 2010).

Our study has several potential limitations. We did not account for maternal smoking during pregnancy; however, such prevalence in this population was quite low. Another serious limitation is the study’s lack of data on the children’s diet and physical activity. Even though both are influential predictors of body weight, there is no evidence to suggest an association between prenatal lead exposure and children’s physical activity and/or dietary patterns. Thus, the lack of control for these parameters could be anticipated to decrease our ability to explain the total variance in child weight over time, but we believe this was unlikely to have introduced confounding. Also, we did not consider the effect of other environmental pollutants on growth and their potential interactions with lead. However, despite these limitations, we believe our results are still valid given the longitudinal design of our study, the adjustment for several predictors and potential confounders, and the use of bone lead as a measure of cumulative prenatal lead exposure. Moreover, our results are generalizable to children from women of reproductive age with levels of lead similar to those of the mothers in our study population, comprising a large age range (15–44 years) and derived from a population with diverse socioeconomic status (low to middle income).

## Conclusion

Prenatal lead, reflected by maternal patella lead, is associated with a lower weight trajectory among 0- to 5-year-old female, but not male, offspring. Given that these effects were confined to an assessment of lead in one bone compartment and in females, results need to be confirmed in other studies.
